# Human Defensin-5 Blocks Ethanol and Colitis-Induced Dysbiosis, Tight Junction Disruption and Inflammation in Mouse Intestine

**DOI:** 10.1038/s41598-018-34263-4

**Published:** 2018-11-02

**Authors:** Pradeep K. Shukla, Avtar S. Meena, Vaishnavi Rao, Roshan G. Rao, Louisa Balazs, RadhaKrishna Rao

**Affiliations:** 1 Department of Physiology, University of Tennessee Health Science Center, Memphis, TN 38103 USA; 2 Department of Pathology, University of Tennessee Health Science Center, Memphis, TN 38103 USA

## Abstract

Alcohol consumption has been shown to cause dysbiosis, but the mechanism involved in it is unknown. Recurrent colitis is known to induce expression of α-defensins in the colon, but the effect of alcohol consumption on it is not known. We investigated the effect of ethanol on α-defensin expression in the small intestine and colitis-induced expression in colon in mice. Furthermore, we evaluated the effect of human defensin-5 (HD5) on ethanol and colitis-induced gut barrier dysfunction and mucosal damage. Recurrent colitis was induced by feeding dextran sulfate sodium (DSS), 3 cycles of 5-days each with 15 days intervals, followed by 30-days remission. Ethanol was fed during the intervals and recovery in a liquid diet with or without HD5. Expression of α-defensins, tight junction (TJ) integrity and cytokine/chemokine expression were analyzed. Chronic ethanol feeding reduced α-defensin expression in the small intestine and colitis-induced defensin expression in the colon. HD5 attenuated the growth of enterotoxigenic *Bacteriodes fragilis* and *E. coli*, but had no effect on non-toxigenic *Bacteriodes fragilis* or probiotics, the *Lactobacilli*. Ethanol and colitis elevated *Enterobacteriaceae*, *Firmicutes* and *Firmicutes* to *Bacteriodetes* ratio in colonic mucosa. HD5 feeding attenuated ethanol and colitis-induced dysbiosis, disruption of intestinal epithelial TJ, mucosal inflammation, expression of pro-inflammatory cytokines and chemokines in the small intestine and colon, and endotoxemia. These results demonstrate that ethanol suppresses intestinal α-defensin expression, leading to dysbiosis, barrier dysfunction, inflammation and endotoxemia. HD5 feeding attenuates intestinal injury caused by ethanol and colitis, indicating that defensin expression is a potential target for treatment of alcoholic tissue injury and colitis.

## Introduction

Enteric α-defensins are antibacterial peptides produced and secreted by the Paneth cells in the small intestine^1^. Human intestine secretes two types of α-defensins, human defensin-5 (HD5) and human defensin-6 (HD6). The rodent α-defensins, also known as cryptdins, consist of many isoforms. While 6 cryptidin proteins (Cryptidin 1–6) have been isolated, more than 25 cryptidin related transcripts have been described^2^. The regulation of expression and secretion of α-defensins are poorly understood. Defensin expression is low at birth and upregulated during the neonatal period^3^, and it is down regulated at old age^4^. In Crohn’s disease, a type of inflammatory bowel disease (IBD), HD5 and HD6 expression in the intestinal Paneth cells is compromised^5^, but, it is over expressed in patients with necrotizing enterocolitis^6^. HD5 expression is upregulated by indomethacin-induced intestinal injury^7^. Colon under normal physiologic conditions does not express α-defensins due to lack of Paneth cells^8^. But, its expression is induced in metaplastic Paneth cells in the colon of IBD patients and in experimental colitis, which is likely a defense mechanism^9,10^. However, the mechanism involved in this induced expression of defensin in colon is unknown. Therefore, further studies are warranted to understand the regulation of α-defensin expression in the intestine under physiologic and pathophysiologic conditions.

The clinical and experimental evidence indicates that chronic alcohol consumption disrupts intestinal epithelial tight junctions (TJ) and adherens junctions (AJ), leading to barrier dysfunction and endotoxemia^11^. TJ forms the physical barrier to the diffusion of macromolecules, including bacterial toxins such as lipopolysaccharide (LPS). TJ is composed of transmembrane proteins such as occludin, claudins, junctional adhesion molecules and tricellulin, which interact with the intracellular adapter proteins, such as zonula occludens (ZO)-1, ZO-2 and ZO-3^12^. AJ is composed of the transmembrane protein, E-cadherin, that interacts with catenins^13^. Disruption of TJ leads to endotoxemia and tissue damage. The mechanism of EtOH-induced junction disruption is poorly understood. Ethanol (EtOH)-induced dysbiosis is likely a contributing factor. Chronic alcohol consumption is known to increase *Firmicutes* to *Bacteriodetes* ratio^14^. The mechanism of EtOH-mediated dysbiosis is unknown. Defensin deficiency is suggested to cause dysbiosis in Crohn’s disease^15^. Paneth cell disruption induces dysbiosis and is linked to many diseases such as Type II diabetes, metabolic syndrome, atherosclerosis, non-alcoholic steatohepatitis, autism, liver cirrhosis and liver cancer. However, the mechanisms involved in defensin expression under physiologic and pathophysiologic conditions are unknown.

In the present study, we conducted investigations to answer the questions whether alcohol consumption affects defensin expression in small intestine in healthy mice and defensin expression in the colon of mice with experimental colitis. We also conducted studies to determine whether HD5 feeding ameliorates alcohol and colitis-induced dysbiosis, disruption of intestinal epithelial junctions, inflammation and endotoxemia.

## Materials and Methods

### Chemicals

Dextran sulfate sodium (DSS) was purchased from MP-Biomedicals (Santa Ana, CA). Hoechst 33342 dye was purchased from Life technologies (Grand Island, NY). AlexaFluor 488-phalloidin (Cat# A12379) was purchased from Thermo Fisher Scientific. EtOH (proof 200, molecular biology grade; Cat# E7023) was purchased from Sigma-Aldrich (St Louis, MO). All other chemicals were either from Sigma-Aldrich (St Louis, MO) or Fisher Scientific (Tustin, CA).

### Antibodies

Anti-Defensin, alpha 6, (DEFA6; Cat# ABIN797137), were purchased from antibodies-online Inc. (Atlanta, GA). Anti-E-cadherin (Cat# 610182), anti-β-catenin (Cat# 6734), anti-occludin (Cat# 331500) and anti-ZO-1 (Cat# 617300) antibodies were purchased from Invitrogen. Cy3-conjugated anti-rabbit IgG (Cat# C2306) was purchased from Sigma-Aldrich. AlexaFluor 488-conjugated anti-mouse IgG (Cat# A11029) was purchased from Thermo Fisher Scientific.

### Bacterial culture

Enterotoxigenic *Bacteroides fragilis* (ETBF) and Non-toxigenic *Bacteroides fragilis* (NTBF) cells were from Dr. Cynthia Sears (Johns Hopkins University) and *E. coli* (ATCC 25922) was from ATCC. ETBF and NTBF were grown in Brain-Heart Infusion (BHI) Broth (37 g/L) containing yeast extract (5 g/L), L-Cysteine (0.5 g/L), clindamycin (6 µg/ml), Hemin (5 mg/L) and Vitamin K (4.9 mg/L) statically at 37 °C in an anaerobic condition for 2–3 days. *E. coli* was grown in Luria-Bertani (LB) medium at 37 °C overnight at 200 rpm. *L. casei* and *L. plantarum* were grown in De Man, Rogosa and Sharpe (MRS) broth for standing overnight at 30 °C.

### HD5 synthesis and oxidative folding

HD5 was custom-synthesized by Biomatik Inc. (Wilmington, DE) and the peptide was purified by HPLC and authenticated by LC-MS/MS analysis. The peptide was dissolved in 8 M GuHCl containing 3 mM reduced and 0.3 mM oxidized glutathione, followed by dilution using 0.25 M NaHCO_3_ to adjust pH to 8.3 and incubated overnight for folding at room temperature. The working concentrations of HD5 and GuHCL were 0.5 mg/ml and 2 M, respectively.

### HD5 antibacterial activity

Synthetic HD5 was tested for antibacterial activity against *Escherichia coli* ATCC 25922, ETBF, NTBF, *L. casei* and *L. plantarum*. The bacteria were grown to mid-logarithmic phase in respective medium, and then diluted to 1 × 10^6^ cfu/mL in 10 mM potassium phosphate in 1% respective medium, pH 7.4. Cells (100 μL) were incubated in the presence of different concentrations of HD5 for 3 h at 37 °C. The cells were then diluted serially in the same buffer, plated on respective medium agar plates and incubated for 18–24 h at 33 °C, and the colonies were counted.

### Animals and diets

Female C57BL/6 mice (12–14 weeks, Harlan Laboratories, Houston, TX) were used for all experiments. All animal experiments were performed according to the protocol approved by the University of Tennessee Health Science Center-Institutional Animal Care and Use Committee. Animals were housed in institutional animal care facility with 12-h light and dark cycles and had free access to regular laboratory chow and water until the start of experiments. Lieber DeCarli diet (Dyet # 710260) was purchased from Dyets Inc. (Bethlehem, PA) and maltodextrin was from Bioserv (Flemington, NJ).

### Recurrent colitis and EtOH feeding

Mice received DSS (3% *w*/*v*) in drinking water; three 5-day courses with 15-day intervals for recovery from each colitis cycle. Colitis was persistent at 30 days after the third DSS cycle. During the 15-day intervals and during the 30 days after 3rd DSS cycle, animals were fed Lieber-DeCarli liquid diet with or without 4% EtOH and with or without HD5; non-EtOH group were pair fed an isocaloric diet (adjusted with maltodextrin). For recurrent colitis studies, mice were grouped as DSS, DSS + EtOH, DSS + EtOH + HD5 (n = 10), and the controls included pair-fed control and EtOH control (n = 5).

### Microbiome analysis

Sections of distal colon (1 cm) were harvested and mucosal DNA was extracted with TRIzol (Invitrogen, Carlsbad, CA) according to the manufacturer’s instructions. DNA was analyzed by qPCR for different bacterial phyla or species using SYBR Green/ROX master mix (Qiagen) in an Applied Biosystems QuantStudio 6 FlexReal-Time PCR instrument. (Norwalk, CT, USA). Primer sequences for 16S ribosomal RNA genes for *Bacteroidetes*, *Firmicutes*, *Enterobacteriaceae*, *E. coli* and *Eubacteria* (Universal) were chosen according to previous publication^16^, and are shown in the Table S1. *Firmicutes* to *Bacteriodetes* ratio was calculated.

### RNA extraction and RT-qPCR

RNA was isolated from colon by using TRIzol kit (Invitrogen, Carlsbad, CA) and quantified using NanoDrop. Total RNA (1.5 μg) was used for generation of cDNAs using the ThermoScript RT-PCR system for first strand synthesis (Invitrogen). Quantitative PCR (qPCR) reactions were performed using cDNA mix (cDNA corresponding to 35 ng RNA) with 300 nmoles of primers in a final volume of 25 μl of 2× concentrated RT2 Real-Time SYBR Green/ROX master mix (Qiagen) in an Applied Biosystems QuantStudio 6 Flex Real-Time PCR instrument (Norwalk, CT, USA). The cycle parameters were: 50 °C for 2 min, one denaturation step at 95 °C for 10 min and 40 cycles of denaturation at 95 °C for 10 s followed by annealing and elongation at 60 °C. Relative gene expression of each transcript was normalized to GAPDH using the ΔΔCt method. Sequences of primers used for qPCR are provided in the supplemental information (Table S1).

### Immuno-fluorescence microscopy

Colon was examined for DEFA6, F-actin, TJ proteins occludin and ZO1 and AJ proteins E-cadherin and β-catenin by confocal microscopy. Cryo-sections of distal colon (10 μm thickness) were fixed in acetone:methanol mixture (1:1) at 20 °C for 2 min and rehydrated in phosphate buffered saline (PBS). Sections were permeabilized with 0.2% Triton X-100 in PBS for 15 min and blocked in 4% non-fat milk in TBST (20 mM Tris, pH 7.2 and 150 mM NaCl). It was then incubated for 1 h with primary antibodies (mouse monoclonal anti-lysozyme, rabbit polyclonal anti-DEFA6, anti-ZO1, anti-β-catenin, and mouse monoclonal anti-E-cadherin and anti-occludin antibodies; all at 1:100 dilution) followed by incubation with secondary antibodies (Cy3-conjugated anti-rabbit IgG antibodies at 1:100 dilution; Molecular Probes, Eugene, OR) and co-stained with AlexaFluor 488-conjugated phalloidin and Hoechst 33342 for 1 h. The fluorescence was examined by using a confocal microscope (Zeiss 710) and images from x-y sections (1 μm) were captured using Zen software under identical conditions of gain and laser. Images for all samples were stacked using the Image J software (NIH, Bethesda, MD) and processed by Adobe Photoshop under identical conditions of brightness and contrast (Adobe Systems Inc., San Jose, CA). In some cases, the fluorescence densities in the epithelial cells were measured using Image J software and data presented as arbitrary units. In lysozyme-stained intestinal sections, number of lysozyme-positive cells in the crypts were counted.

### Histopathology

Distal colon was fixed in 10% buffered formalin and 8 μm thick paraffin embedded sections were stained with hematoxylin and eosin. Stained sections were imaged in a Nikon 80Ti microscope using 10X objective lens and a color camera. Four to six samples were harvested from segments of colon and fixed in buffered formalin (10%). Fixed tissues were processed and embedded by routine histology protocols. The 4 μm thick sections were deparafinized and stained with Hematoxylin-Eosin stain. The blinded histological evaluation was performed by a board certified anatomic pathologist being familiar with the patho-histology of IBD. The most characteristic histopathological hallmarks of IBD were evaluated using a modified recommendation (*Nature Communications* ISSN 2041-1723 online). The histological changes were graded as no abnormality (score 0), mild (score 1), moderate (score 2), and severe changes (score 3).

### Plasma endotoxin assay

Plasma endotoxin concentrations were measured using Pierce LAL Chromogenic Endotoxin Quantitation Kit (Thermo Scientific, Cat# 88282) according to vendor’s instructions.

### Plasma cytokine assay

Plasma cytokine levels were measured using commercially available immunoassay ELISA kits for mice (R&D System, Minneapolis, MN, USA). IL-1β (Cat# DY401), TNF-α (Cat# DY410) and interleukin-6 (Cat# DY406) levels were estimated according to the manufacturer’s instructions. The results are expressed as picograms of cytokine per milliliter of plasma.

### Statistical Analyses

All data are expressed as Mean ± SEM. The differences among multiple groups were first analyzed by ANOVA (Prism 6.0). When a statistical significance was detected, Tukey’s *t* test was used to determine the statistical significance between multiple testing groups and the corresponding control. Statistical significance was established at 95%.

All authors had access to the study data and had reviewed and approved the final manuscript.

## Results

### Chronic EtOH Feeding down regulates α-defensin expression in mouse intestine

**Figure 1:**
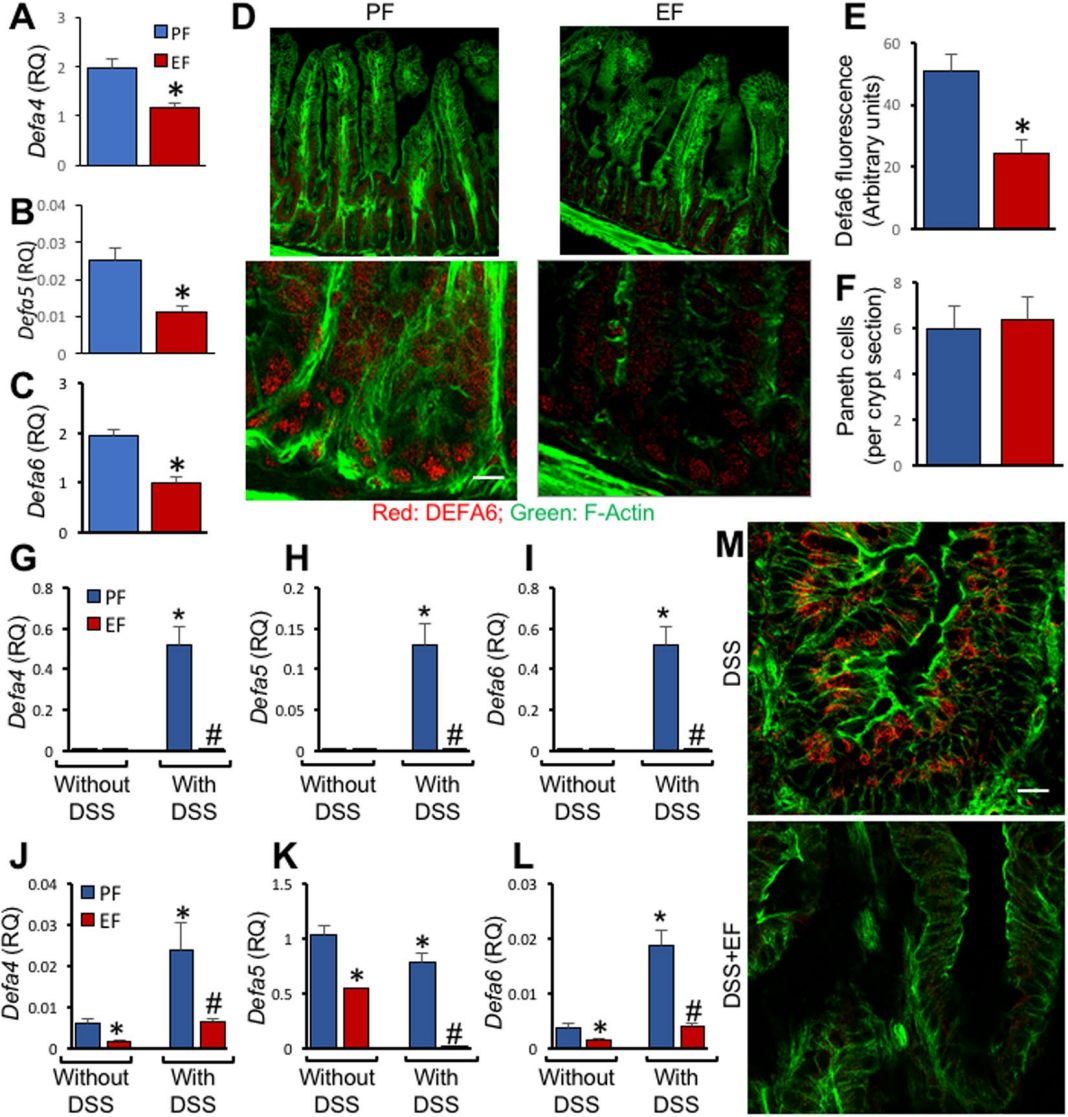
Chronic EtOH feeding down regulates α-defensin expression in mouse intestine. (**A**–**F**) Adult mice were fed a liquid diet with EtOH (EF) or isocaloric maltodextrin (PF) for 4 weeks. RNA extracted from ileal mucosa was subjected to RT-qPCR for *Defa4* (**A**), *Defa5* (**B**) and *Defa6* (**C**) genes. Cryosections of ileum from PF and EF groups were stained for F-actin (green) and DEFA6 (**D**). Density of DEFA6 fluorescence was measured (**E**) and number of lysozyme-positive cells at the crypt base were counted (**F**). Values are mean ± sem (n = 4). Asterisks indicate the value that is significantly (p < 0.05) different from corresponding PF value. (**G**–**M**) In EtOH and recurrent colitis model, adult mice were subjected to DSS-induced colitis three times with 15-day recovery intervals, followed by 15-day recovery from third colitis. During the intervals and recovery period, mice were fed a liquid diet with EtOH (EF) or isocaloric maltodextrin (PF). RNA extracted from mucosa of colon (**G**–**I**) and ileum (**J**–**L**) was subjected to RT-qPCR for *Defa4* (**G**,**J**), *Defa5* (**H**,**K**) and *Defa6* (**I**,**L**) genes. Cryosections of colon from DSS and DSS + EtOH groups were stained for F-actin (green) and DEFA6 (**M**); the white bar represents 8 μm in distance. Values in panels A–C and E–J are mean ± sem (n = 6). Asterisks indicate the values that are significantly (p < 0.05) different from corresponding PF values, and hash tags indicate the values that are significantly different from corresponding DSS values. The white bars in panels D and M represent 5.8 μm distance.

To determine the effect of EtOH consumption on intestinal expression of α-defensins, mice were fed Lieber-DeCarli liquid diet with EtOH or isocaloric maltodextrin. RNA samples extracted from the mucosa of ileum were analyzed for expression of α-defensins by RT-qPCR. EtOH feeding significantly reduced mRNA for *Defa4, Defa5* and *Defa6* genes (Fig. 1A–C). Confocal immunofluorescence microscopy showed the predominant localization of DEFA6 in the crypt epithelial cells in the ileum of pair fed mice, but it is dramatically reduced in the ileum of EtOH-fed mice (Fig. 1D). The DEFA6 fluorescence density in crypt cells was significantly reduced in EtOH-fed mice (Fig. 1E), but the number of lysozyme-positive cells at the base of crypts was not altered (Fig. 1F). Defensin expression was absent in the colon of healthy control mice, and EtOH feeding did not induce α-defensin expression in colon (Fig. 1G–I). However, colitis is known to induce α-defensin expression in colon. Our data show that DSS-induced colitis induces expression of α-defensins in mouse colon (Fig. 1G–I). Interestingly, chronic EtOH feeding abolished colitis-induced expression of α-defensins in colon. DSS-induced colitis also elevated expression of *Defa4* and *Defa6* genes in the ileum (Fig. 1J–L). EtOH feeding caused dramatic reduction in the expression of *Defa4, Defa5* and *Defa6* genes in the ileum of DSS-treated mice. Confocal immunofluorescence microscopy showed the predominant localization of DEFA6 in the crypt epithelial cells in the colon of DSS-treated mice, but it was very low in the colon of EtOH-fed DSS-colitis mice (Fig. 1M).

### Synthetic human defensin-5 (HD5) exhibit antibacterial activity

**Figure 2:**
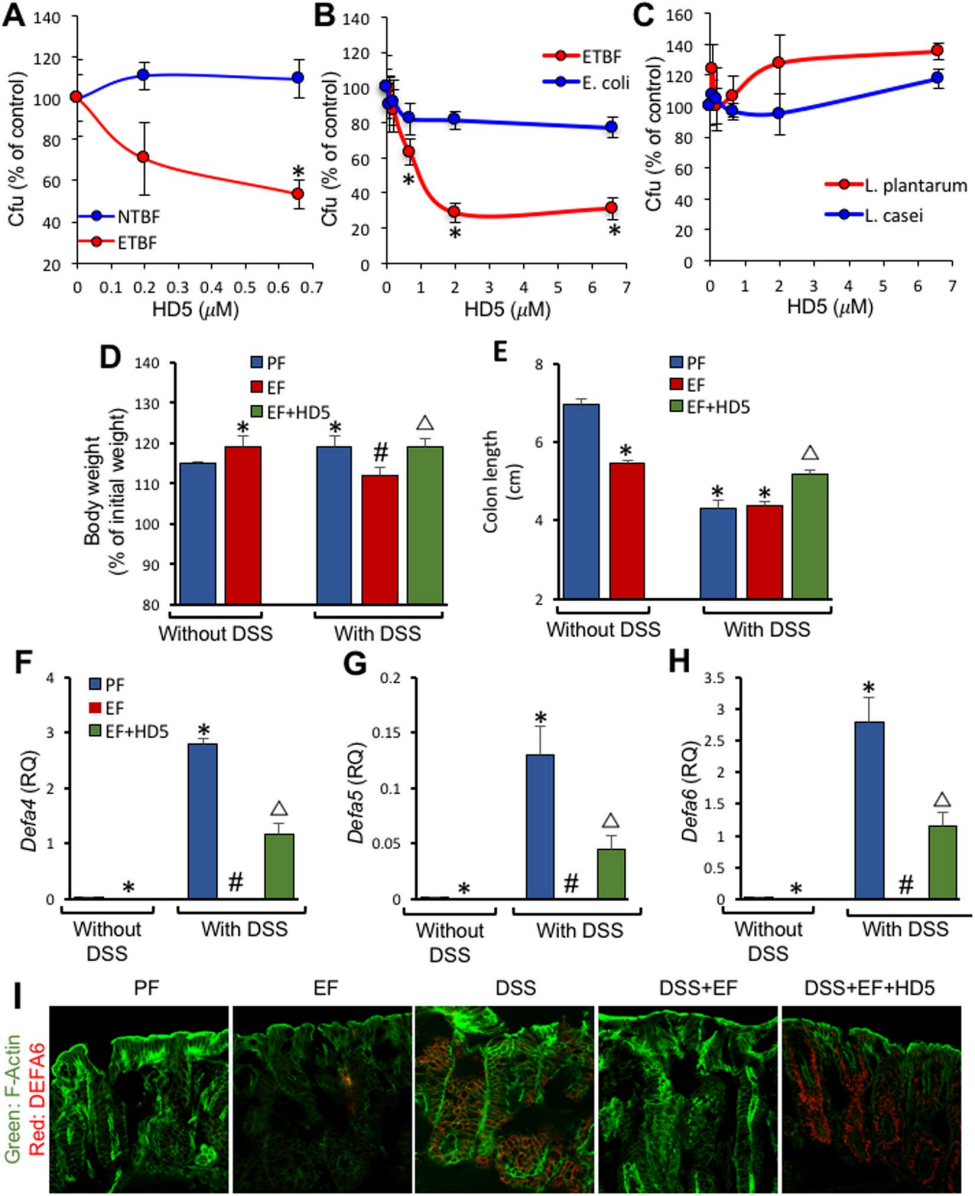
Effect of synthetic HD5 on DSS-induced recurrent colitis and EtOH feeding. (**A**–**C**) The antibacterial activity of synthetic HD5 was evaluated in Non-toxigenic NTBF and entero-toxigenic ETBF (**A**), ETBF and *E. coli* (**B**) or *L. casei* and *L. plantarum* (**C**). Bacterial cultures were incubated with different concentrations of HD5 followed by growth on agar plates and counted the colony forming units (cfu). Values are mean ± sem (n = 4). In panels A and B, asterisks indicate the values that are significantly (p < 0.05) different from corresponding NTBF (**A**) or ETBF (**B**) values. (**D**,**I**) Adult female mice were subjected to DSS-induced colitis three times with 15-day recovery intervals. During the recovery intervals mice were fed a liquid diet with EtOH (EF) or isocaloric maltodextrin (PF). Body weights (**D**) and colon lengths (**E**) were measured. RNA extracted from mucosa of colon was subjected to qPCR for *Defa4* (**F**), *Defa5* (**G**) and *Defa6* (**H**) genes. Cryosections of colon were stained for F-actin (green) and Defa6 (red) and the fluorescence imaged by confocal fluorescence microscopy (**I**). Values in panels D-H are mean ± sem (n = 6). Asterisks indicate the values that are significantly (p < 0.05) different from corresponding PF values, and the hash tags indicate the values significantly different from corresponding values for DSS without EtOH group. The symbol Δ indicates values that are different from corresponding values for DSS + EF group.

Human intestine expresses two α-defensins, HD5 and HD6. HD5 exhibit high antibacterial activity. In order to determine the effect of HD5 on EtOH and colitis-mediated intestinal injury we synthesized HD5. The synthetic HD5 was purified by high performance liquid chromatography (Fig. S1) and the structure was authenticated by mass spectrometric analysis and microsequencing (Fig. S2). The antibacterial activity was confirmed by evaluating its effect on growth of *Bacteriodes fragilis*, *E. coli* and probiotics. HD5 dose-dependently reduced the growth of enterotoxigenic ETBF, but not non-toxigenic NTBF (Fig. 2A). HD5 dose-dependently reduced the growth of *E. coli*, however, it was much less potent on *E. coli* compared to its effect on ETBF (Fig. 2B). HD5, up to 6.66 μM, did not affect the growth of probiotics, the *L. plantarum* and *L. casei* (Fig. 2C).

### Effect of HD5 feeding on colitis-induced defensin expression

Ulcerative colitis is a recurrent relapsing inflammatory disease of colon. In our animal model, we induced colitis for 3 cycles with 15-day intervals and 30-day recovery after the third colitis cycle. Liquid diets with or without EtOH and with or without HD5 were fed during the intervals and the final recovery period. EtOH feeding and colitis significantly increased body weights of mice, however, when EtOH was fed to mice with colitis the body weights were significantly reduced. HD5 feeding blocked the combined effect of EtOH and colitis on body weights (Fig. 2D). Colon lengths were significantly reduced by EtOH and colitis. Feeding EtOH to mice with colitis did not further decrease the colon lengths. But, HD5 significantly attenuated the reduction of colon length (Fig. 2E).

The expression of *Defa4, Defa5* and *Defa6* genes was induced by colitis in the colon and EtOH feeding abolished this effect of colitis (Fig. 2F–H). HD5 feeding partially blocked the effect of EtOH on colitis-induced defensin expression. This observation was confirmed by immunofluorescence staining for DEFA6. DEFA6 was detected in the colon of DSS-treated mice and was found to be localized predominantly in the crypt regions of colonic epithelium (Fig. 2I). EtOH feeding abolished DEFA6 staining; but, this effect of EtOH was absent in HD5-fed mouse colon.

**Figure 3:**
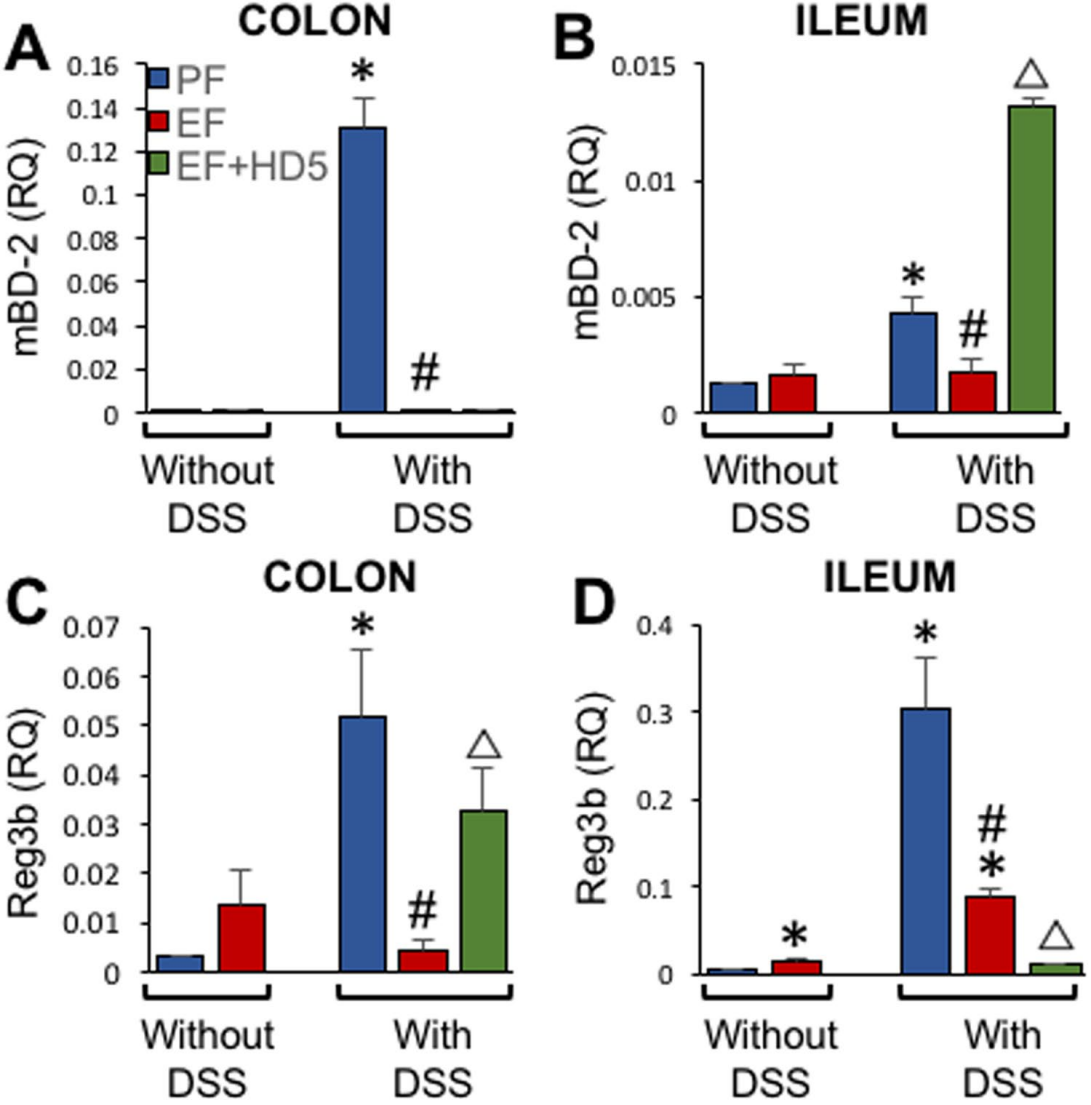
Effect of EtOH and colitis on expression of epithelial antibacterial peptides. Adult mice were subjected to DSS-induced colitis three times with 15-day recovery intervals. During the recovery intervals mice were fed a liquid diet with EtOH (EF) or isocaloric maltodextrin (PF). RNA extracted from mucosa of colon (**A**,**C**) and ileum (**B**,**D**) was subjected to qPCR for *mBD-2* (**A**,**B**) and *Reg3b* (**C**,**D**) genes. Values are mean ± sem (n = 6). Asterisks indicate the values that are significantly (p < 0.05) different from corresponding PF values, and the hash tags indicate the values significantly different from corresponding values for DSS without EtOH group. The symbol Δ indicates values that are different from corresponding values for DSS + EF group.

We measured the mRNA for non-Paneth cell defensins, β-defensin-2 (mBD-2), mBD-3 and Reg3b, in ileum and colon. EtOH failed to alter the level of mBD-2 mRNA in colon or ileum, but it blocked colitis-induced expression of mBD-2 both in colon (Fig. 3A) and ileum (Fig. 3B). HD5 treatment elevated mBD-2 mRNA in the ileum. Reg3b mRNA in colon and ileum was elevated by EtOH and colitis (Fig. 3C,D), but EtOH significantly reduced colitis-induced elevation of Reg3b mRNA. HD5 treatment had opposing effects in colon and ileum, while it elevated Reg3b mRNA in colon, it was reduced by HD5 in the ileum. Levels of mBD-3 mRNA are negligibly low in all groups.

### HD5 blocks EtOH and colitis-induced bacterial dysbiosis in colonic mucosa

**Figure 4:**
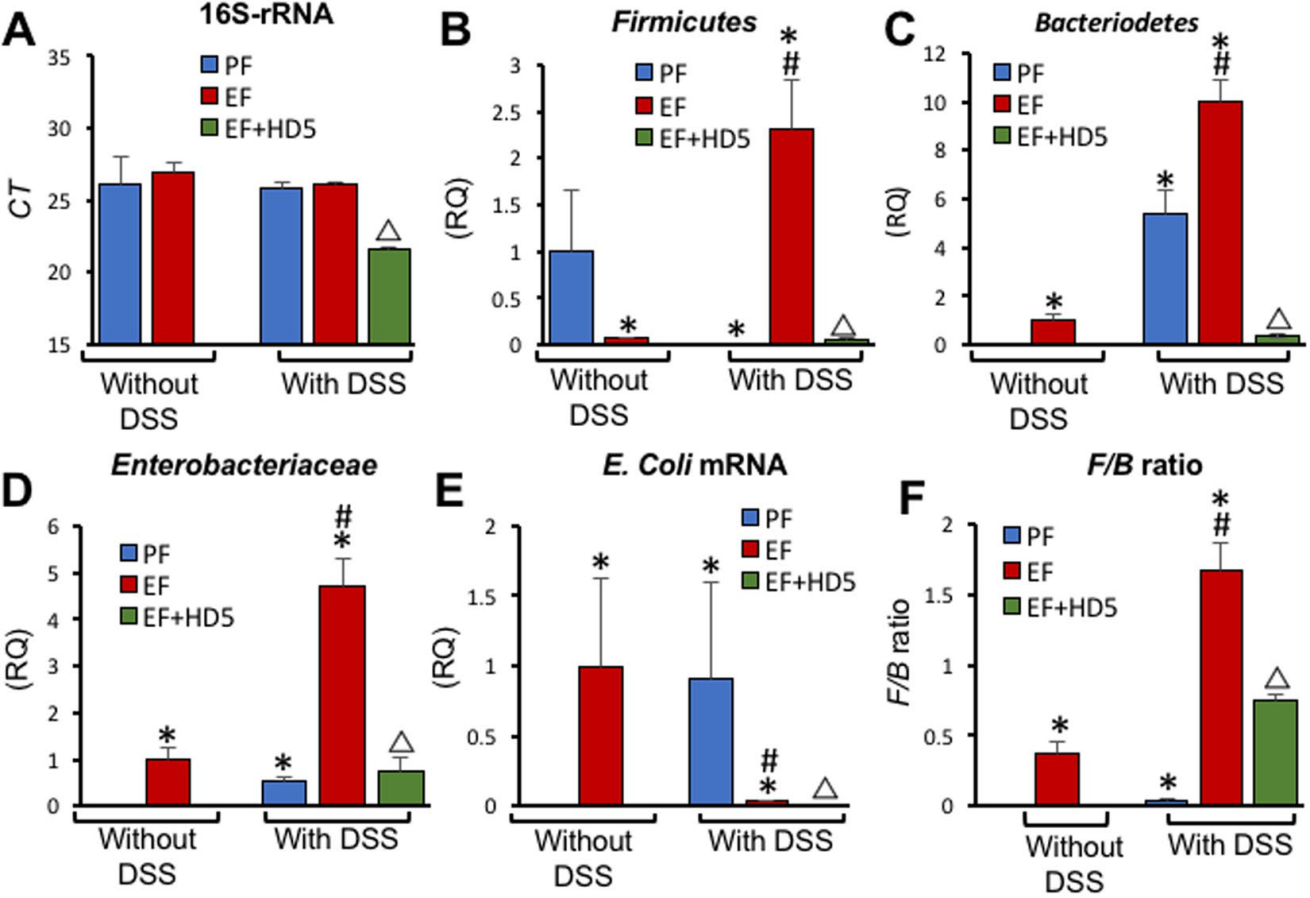
HD5 attenuates DSS-colitis and EtOH-induced bacterial dysbiosis in colonic mucosa. Adult mice were subjected to DSS-induced colitis three times with 15-day intervals, followed by 30-day recovery from third colitis. During the intervals and recovery period, mice were fed a liquid diet with EtOH (EF) or isocaloric maltodextrin (PF) and with or without HD5 supplementation. DNA extracted from colonic mucosa was subjected to qPCR for 16 S ribosomal RNA (**A**), *Firmicutes* (**B**), *Bacteriodetes* (**C**), *Enterobacteriaceae* (**D**) and *E. coli* (**E**). *Firmicutes* to *Bacteriodetes* (F/B) ratio was calculated (**F**). Values are mean ± sem (n = 6). Asterisks indicate the values that are significantly (p < 0.05) different from corresponding PF values, and the hash tags indicate the values significantly different from corresponding values for DSS without EtOH group. The symbol Δ indicates values that are different from corresponding values for DSS + EF group.

The overall 16S rRNA levels in the colon of EtOH, DSS or EtOH-fed and DSS-treated mice were not different from each other (Fig. 4A); whereas, HD5 treatment significantly reduced the levels of mucosa-bound 16S rRNA. The levels of *Firmicutes* were very low in colon of EtOH-fed or DSS-treated mice (Fig. 4B). But, the combination of EtOH feeding and DSS-colitis dramatically elevated *Firmicutes* in the colon. HD5 treatment abolished the elevation of *Firmicutes* in the colon of EtOH-fed and DSS-treated mice. EtOH feeding slightly elevated *Bacteriodetes* and significantly enhanced DSS colitis-induced increase in *Bacteriodetes* (Fig. 4C). These effects of EtOH and DSS were blocked by HD5 feeding. EtOH or DSS-colitis alone slightly elevated *Enterobacteriaceae* (Fig. 4D). This effect on *Enterobacteriaceae* was dramatically high when EtOH feeding was combined with DSS colitis, and HD5 treatment blocked this effect. *E. coli* levels were high in mice treated with EtOH or DSS, which was reduced when EtOH feeding and DSS-colitis were combined. HD5 further reduced the level of mucosa-bound *E. coli* (Fig. 4E). *Firmicutes* to *Bacteriodetes* ratio (F/B ratio) in mouse colon was elevated several folds by EtOH feeding and this response was nearly 4-fold higher when EtOH feeding was combined with colitis (Fig. 4F). HD5 significantly reduced F/B ratio in the colonic mucosa of EtOH-fed and DSS-treated mice.

### HD5 feeding prevents EtOH and colitis-induced tight junction disruption in mouse colon

**Figure 5:**
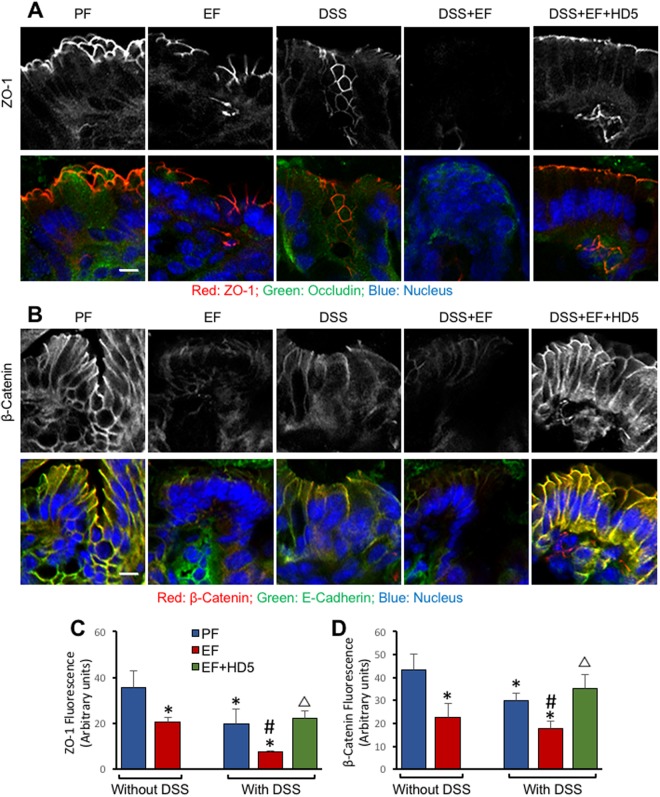
HD5 prevents DSS-colitis and EtOH-induced disruption of tight junction (TJ) and adherens junction (AJ) in mouse colon. Adult mice were subjected to DSS-induced colitis three times with 15-day intervals, followed by 30-day recovery from third colitis. During the intervals and recovery period, mice were fed a liquid diet with EtOH (EF) or isocaloric maltodextrin (PF) and with or without HD5 supplementation. Cryosections of colon were stained for occludin (green) and ZO-1 (red) (**A**) or E-cadherin (green) and β-catenin (red) (**B**) and the fluorescence images captured in a confocal fluorescence microscope. The white bars represent 8 μm in distance. Densities of ZO-1 (**C**) and β-catenin (**D**) fluorescence was measured. Values are mean ± sem (n = 3). Asterisks indicate the values that are significantly (p < 0.05) different from corresponding PF values, and the hash tags indicate the values significantly different from corresponding values for DSS without EtOH group. The symbol Δ indicates values that are different from corresponding values for DSS + EF group.

Staining cryosections of colon showed a co-localization of occludin and ZO-1 at the epithelial junctions (Fig. 5A). EtOH feeding or DSS-induced colitis caused a reduction of junctional stain for occludin and ZO-1, and the loss of stain for these tight junction proteins was more severe when EtOH feeding was combined with colitis. HD5 blocked the effect of EtOH and DSS-induced colitis. Junctional distribution of E-cadherin and β-catenin was also reduced by EtOH feeding and DSS-induced colitis (Fig. 5B). HD5 feeding blocked the effect of EtOH and colitis on junctional distribution of E-cadherin and β-catenin. ZO-1 (Fig. 5C) and β-catenin (Fig. 5D) fluorescence densities measured in the surface epithelial cells confirmed significant reduction of junctional fluorescence by EtOH and colitis. The effects were significantly greater when EtOH feeding was combined with colitis.

### HD5 attenuates EtOH and DSS-induced mucosal inflammation in colon

**Figure 6:**
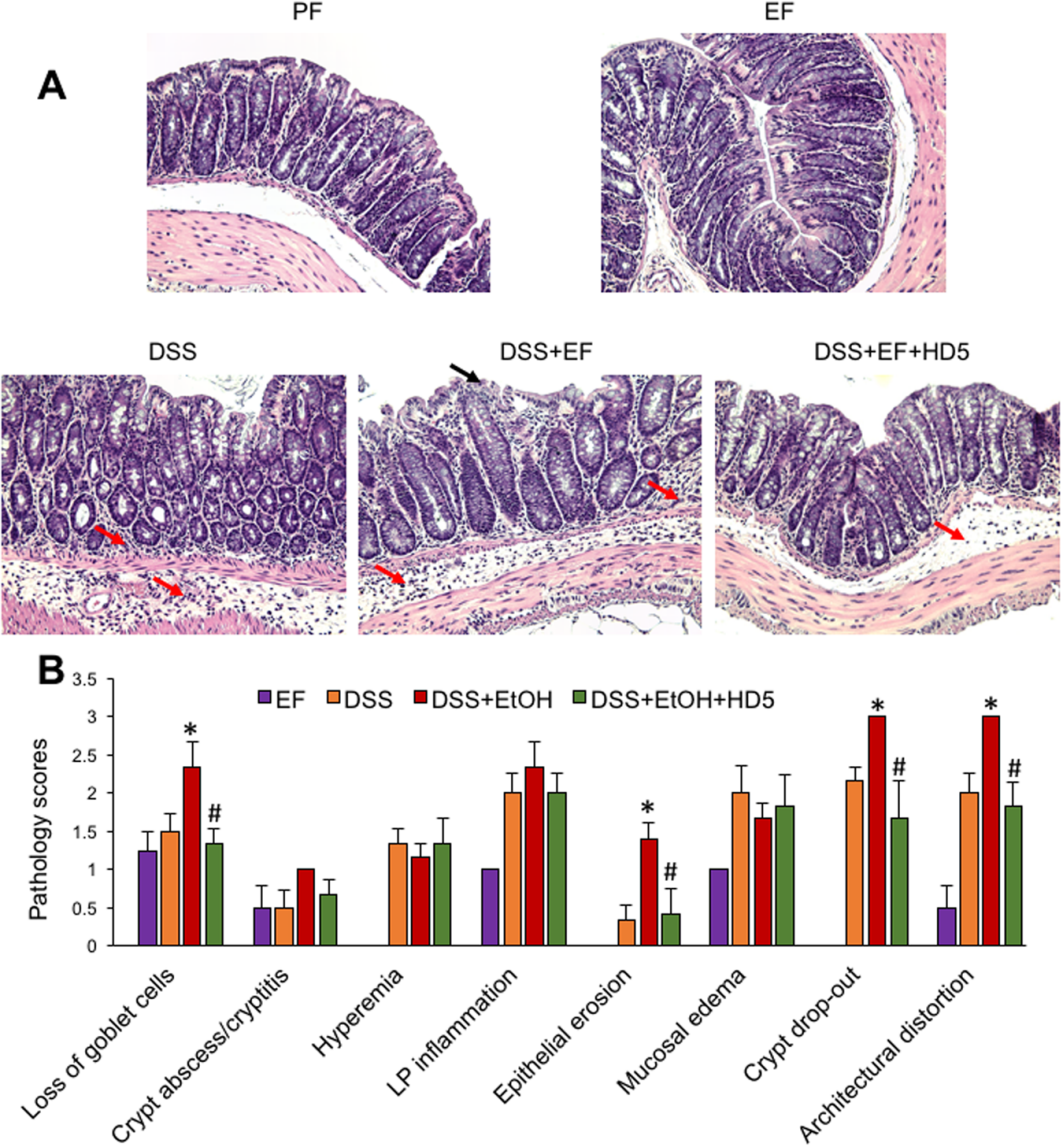
HD5 prevents DSS-induced colitis and EtOH-induced colonic mucosal damage. (**A**) Adult mice were subjected to DSS-induced colitis three times with 15-day intervals, followed by 30-day recovery from third colitis. During the intervals and recovery period, mice were fed a liquid diet with EtOH (EF) or isocaloric maltodextrin (PF) and with or without HD5 supplementation. Paraffin sections of colon were stained with H & E dyes, and the bright field images were captured in a light microscope. Black arrow indicates mucosal damage, and red arrows indicate neutrophil infiltration. (**B**) Slides were scored for various histopathological indicators. Values are mean ± sem (n = 6). Asterisks indicate the values that are significantly (p < 0.05) different from corresponding EF or DSS values, and the hash tags indicate the values significantly different from corresponding values for DSS with EtOH group.

**Figure 7:**
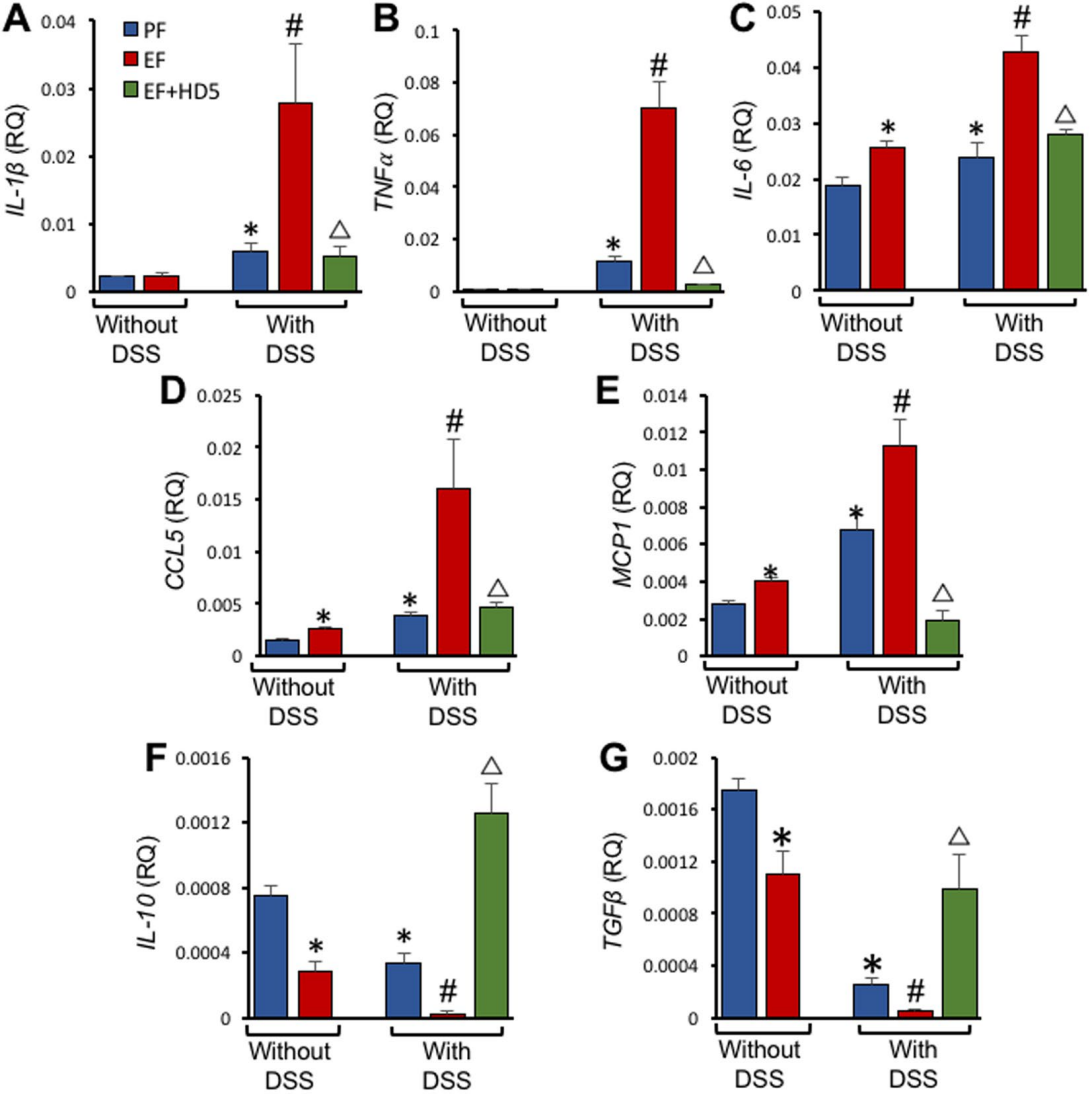
Effect of HD5 on expression of cytokines and chemokines in the colon of DSS-colitis and EtOH-fed mice. Adult mice were subjected to DSS-induced colitis three times with 15-day intervals, followed by 30-day recovery from third colitis. During the intervals and recovery period, mice were fed a liquid diet with EtOH (EF) or isocaloric maltodextrin (PF) and with or without HD5 supplementation. RNA extracted from colonic mucosa was subjected to RT-qPCR for *IL-1β* (**A**), *TNFα* (**B**), *IL-6* (**C**), *CCL*5 (**D**), *MCP1* (**E**), *IL-10* (**F**) and *TGFβ* (**G**) genes. Values are mean ± sem (n = 6). Asterisks indicate the values that are significantly (p < 0.05) different from corresponding PF values, and the hash tags indicate the values significantly different from corresponding values for DSS without EtOH group. The symbol Δ indicates values that are different from corresponding values for DSS + EF group.

Histopathology of colonic sections showed no significant morphological alterations in EtOH-fed mouse colon, whereas neutrophil infiltration was present in the colon of DSS-treated mice (Fig. 6A), which appears to be unaffected by EtOH, but reduced by HD5. Pathologic scoring indicated that both EtOH feeding and DSS-induced colitis are associated with significant loss of goblet cells and architectural distortion (Fig. 6B). This response was significantly higher when EtOH feeding was combined with DSS-colitis, and HD5 feeding significantly reduced these effects. EtOH feeding did not cause epithelial erosion or crypt drop-out in colonic mucosa. However, EtOH enhanced DSS-colitis-induced epithelial erosions and crypt drop-out, and HD5 significantly reduced these effects. EtOH significantly elevated *IL-6* mRNA in colonic mucosa, and DSS-induced colitis elevated mRNA for *IL-1β*, *TNFα* and *IL-6* (Fig. 7A–C). DSS-induced increase of mRNA for these cytokines was further elevated several folds by EtOH feeding, and HD5 feeding effectively blocked these responses to EtOH and DSS. EtOH and DSS-colitis also elevated mRNA for *CCL5* and *MCP1* genes in the colon (Fig. 7D,E). The effect of colitis was elevated several folds by EtOH feeding, and HD5 dramatically reduced EtOH and colitis-mediated expression of *CCL5* and *MCP1*.

On the other hand, both EtOH and colitis significantly reduced the levels of mRNA for *IL-10* and *TGFβ* genes (Fig. 7F,G). A combined EtOH feeding and DSS-colitis almost completely depleted the mRNA levels for these genes. HD5 treatment blocked these effects of EtOH and colitis and maintained *IL-10* and *TGFβ* mRNA at high levels.

### HD5 blocks EtOH and colitis-induced expression of pro-inflammatory cytokines and chemokines in the small intestine

**Figure 8:**
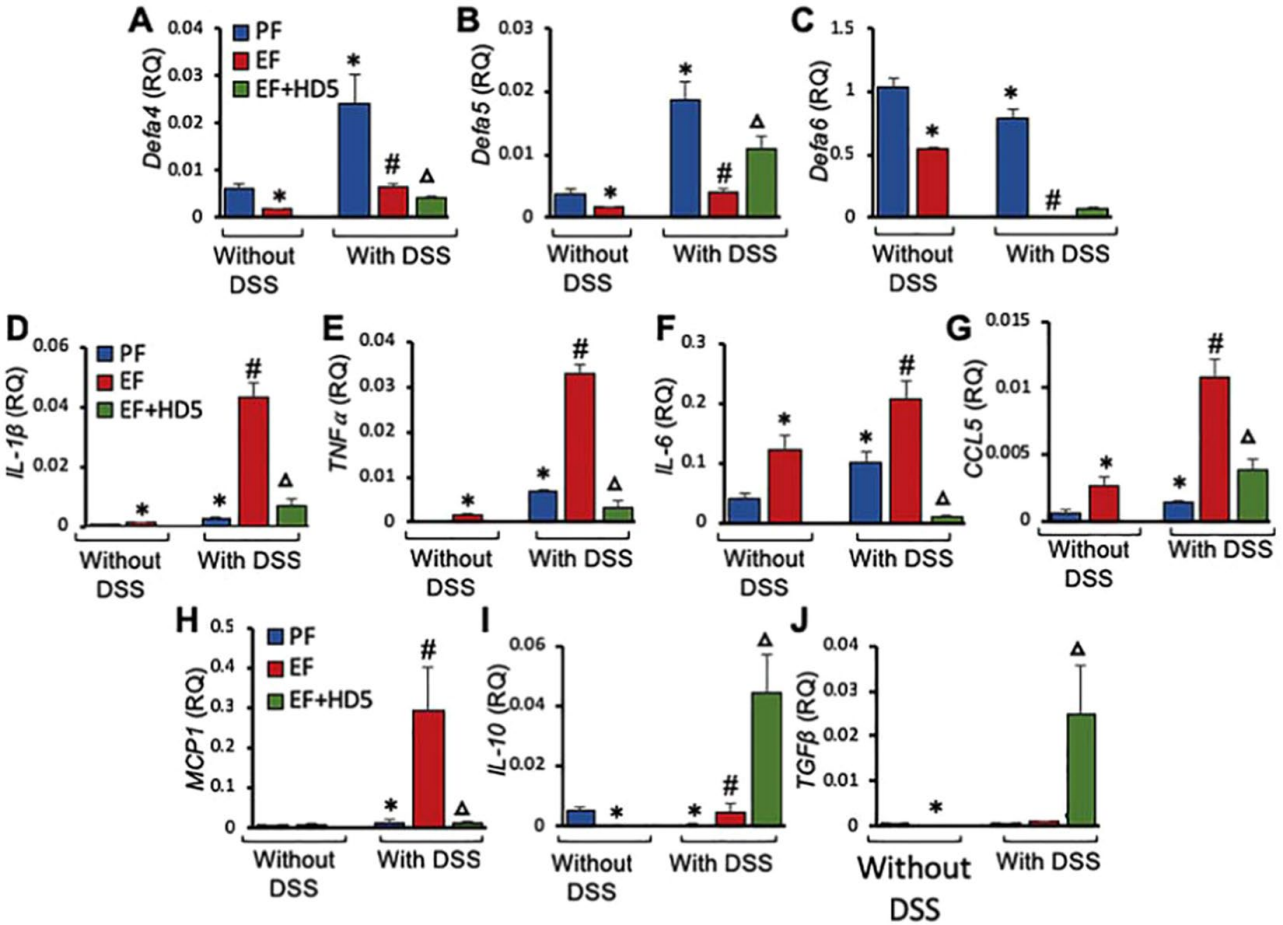
Effect of HD5 feeding on EtOH-induced down regulation of α-defensin, cytokine and chemokine gene expression in mouse ileum. Adult mice were subjected to DSS-induced colitis three times with 15-day intervals, followed by 30-day recovery from third colitis. During the intervals and recovery period, mice were fed a liquid diet with EtOH (EF) or isocaloric maltodextrin (PF) and with or without HD5 supplementation. (**A**–**C**) RNA extracted from ileal mucosa was subjected to RT-qPCR for *Defa4* (**A**), *Defa5* (**B**) and *Defa6* (**C**) genes. (**D**–**J**) RNA extracted from mucosa of ileum was subjected to qPCR for *IL-1β* (**D**), *TNFα* (**E**), *IL-6* (**F**), *CCL*5 (**G**), *MCP1* (**H**), *IL-10* (**I**) and *TGFβ* (**J**) genes. Values in all panels are mean ± sem (n = 6). Asterisks indicate the values that are significantly (p < 0.05) different from corresponding PF values, and the hash tags indicate the values significantly different from corresponding values for DSS without EtOH group. The symbol Δ indicates values that are different from corresponding values for DSS + EF group.

DSS-induced colitis significantly elevated mRNA for *Defa4* and *Defa5*, but not *Defa6* genes in the ileum (Fig. 8A–C). EtOH feeding caused dramatic reduction of colitis-induced expression of *Defa4, Defa5* and *Defa6* genes. HD5 feeding partially blocked the effect of EtOH and DSS-colitis on *Defa5* and *Defa6* mRNA, but the effect on the mRNA for *Defa4* gene was further reduced. EtOH feeding or DSS-induced colitis significantly elevated mRNA for *IL-1β, TNFα and IL-6* genes in ileal mucosa (Fig. 8D–F). DSS-induced elevation of mRNA for these cytokines was increased several folds by EtOH feeding, and HD5 feeding effectively blocked these effects of EtOH and DSS-colitis. EtOH and DSS-colitis also elevated mRNA for *CCL5* and *MCP1* genes (Fig. 8G,H). The effect of colitis was elevated several folds by EtOH feeding, and HD5 reduced EtOH and colitis-mediated expression of *CCL5* and *MCP1*. On the other hand, EtOH and DSS significantly reduced the levels of mRNA for *IL-10* and *TGFβ* genes (Fig. 8I,J). HD5 treatment in EtOH and DSS-treated mice induced a robust elevation of mRNA for *IL-10* and *TGFβ* genes.

**Figure 9:**
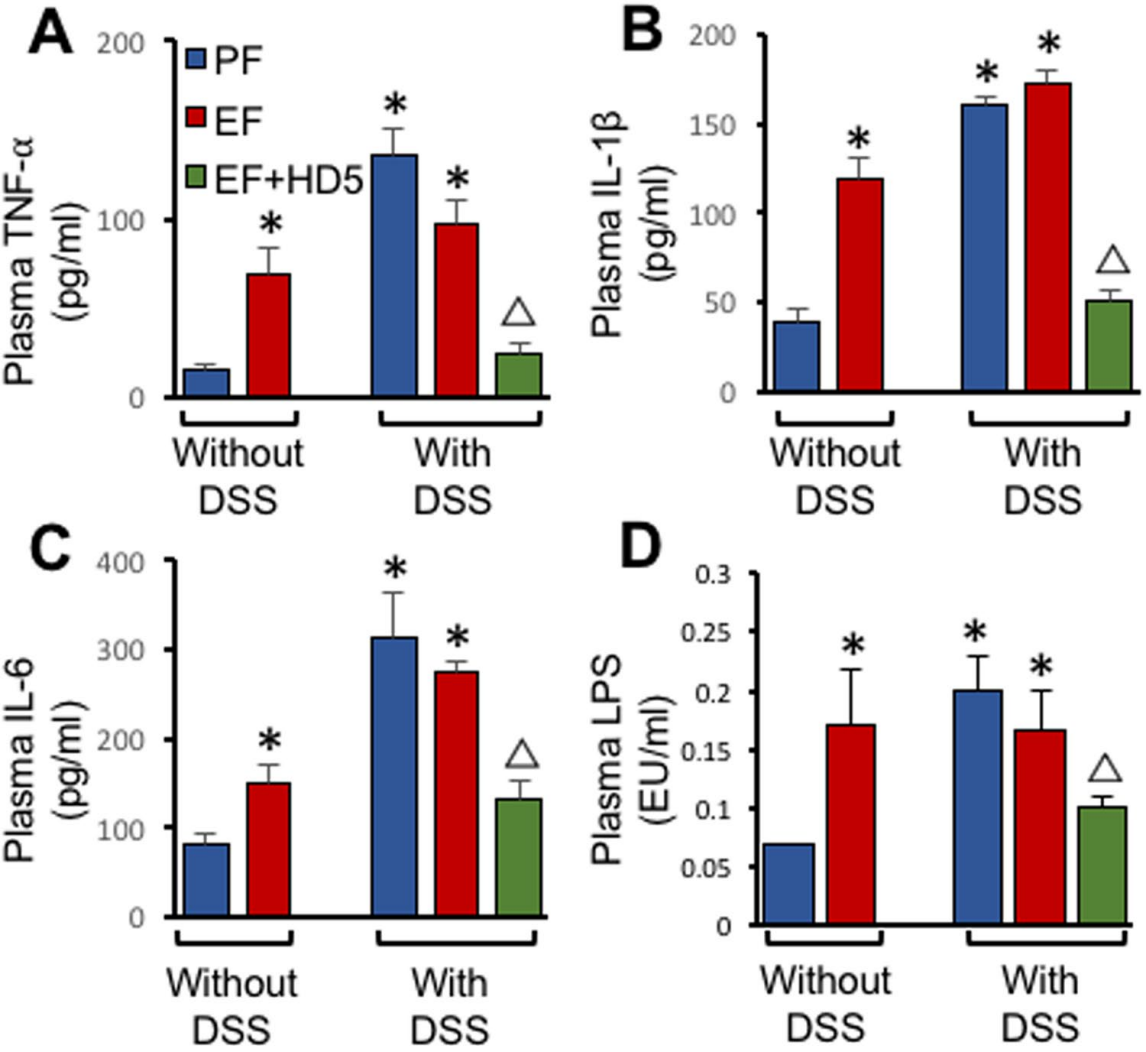
Effect of HD5 on EtOH and colitis-induced upregulation of plasma cytokines. Adult mice were subjected to DSS-induced colitis three times with 15-day intervals, followed by 30-day recovery from third colitis. During the intervals and recovery period, mice were fed a liquid diet with EtOH (EF) or isocaloric maltodextrin (PF) and with or without HD5 supplementation. TNFα (**A**), IL-1β (**B**) and IL-6 (**C**) levels in plasma were measured by ELISA. Plasma samples from mice in different groups were also analyzed for lipopolysaccharide (LPS) levels (**D**). Values in all panels are mean ± sem (n = 6). Asterisks indicate the values that are significantly (p < 0.05) different from corresponding PF values, and the symbol Δ indicates values that are different from corresponding values for DSS + EF group.

Cytokine levels in the plasma was measured by ELISA. Plasma levels of TNFα (Fig. 9A), IL-1β (Fig. 9B) and IL-6 (Fig. 9C) in the plasma were significantly increased by EtOH feeding and colitis. EtOH did not influence colitis-induced elevation of these cytokines in the plasma, but HD5 treatment effectively blocked EtOH and colitis-induced elevation of plasma cytokines.

### HD5 blocks EtOH and colitis-induced endotoxemia

Chronic EtOH feeding significantly elevated plasma LPS levels (Fig. 9D). Similarly, DSS-induced colitis with or without EtOH feeding elevated plasma LPS. HD5 feeding significantly reduced plasma LPS levels in EtOH-fed DSS-colitis mice.

## Discussion

Intestinal α-defensins, the antibacterial peptides, are known to play an innate host defense role against bacterial, fungal and viral infections and maintenance of intestinal mucosal homeostasis^15,17^. While evidence indicates that the defensins are required for the maintenance of intestinal bacterial composition, the factors that regulate the expression of these antibacterial peptides are poorly understood. Furthermore, the potential therapeutic benefits of defensins in the treatment of gastrointestinal diseases has not been tested. In the present study, we provide evidence that alcohol consumption compromises expression of intestinal defensins, and that HD5 prevents alcohol and colitis-induced gut barrier disruption, mucosal inflammation and endotoxemia in mice.

Under normal physiological conditions, α-defensin expression is confined to the small intestine, and colon does not express these antibacterial peptides. However, in the small intestine they are released into the intestinal lumen, which are likely to be delivered into the colonic lumen. Expression of α-defensins are known to be down regulated in Crohn’s disease^5^ and by intestinal microbial metabolites such as lactate^18^. In the present study, our data show for the first time, that chronic EtOH feeding down regulates the expression of *Defa4*, *Defa5* and *Defa6* genes in mouse ileum. Immunofluorescence microscopy confirms the dramatic reduction of DEFA6 in the crypts of ileum by EtOH feeding. The number of lysozyme-positive cells at the crypt base was not altered by EtOH, suggesting that EtOH does not affect Paneth cell differentiation, rather the expression of defensins in Paneth cells is suppressed. Previous studies showed that expression of α-defensins is induced in colon by colitis^9,10^. Results of our present study show that DSS-induced colitis induces *Defa4*, *Defa5* and *Defa6* expression in mouse colon, and that EtOH feeding abrogates the colitis-induced expression of defensins in colon. These results demonstrate that EtOH consumption down regulates the expression of α-defensins in the small intestine as well as colitis-induced expression in the colon. The inflammation-induced expression of defensins in colon is likely an inducible defense mechanism against the inflammation-mediated tissue injury. Alcohol consumption may suppress this defense mechanism and exacerbate inflammation, resulting in more severe mucosal damage. DSS-induced colitis was confined to colon and without any mucosal damage in the small intestine. But, the α-defensin expression in the ileum was upregulated during DSS-induced colitis, and EtOH consumption effectively suppressed the defensin expression in the ileum of mice with DSS-induced colitis.

EtOH-mediated down regulation of α-defensin expression in the small intestine and colitis-induced defensin expression in the colon by EtOH suggested that defensin supplementation might prevent EtOH and colitis-induced intestinal mucosal injury. In the mouse intestine, over 25 cryptidin-encoding transcripts have been described, but, the specific functions of different cryptidin isoforms are poorly understood. On the other hand, human intestinal Paneth cells secrete only two types of α-defensins, HD5 and HD6, with HD5 having a high antibacterial activity^19^. Therefore, we chose HD5 to test its effect on EtOH and colitis-induced intestinal injury. Previous study demonstrated that expression of HD5 in transgenic mice induced resistance to salmonella infection^20^. Additionally, determining the therapeutic value of HD5 is of high clinical relevance to humans. We synthesized HD5, and its antibacterial activity was confirmed in few bacterial species. Synthetic HD5 reduced the growth of enterotoxigenic ETBF, but not non-pathogenic NTBF. HD5 also reduced the growth of *E. coli*, but less effectively than its effect on ETBF. HD5 on the other hand, had no effect on the probiotics, *L. casei* and *L. plantarum*. Therefore, our data show that the antibacterial activity of HD5 is more selective on pathogenic bacteria.

To determine the intestinal mucosal protective effect of HD5 we used a mouse model of recurrent colitis and chronic EtOH feeding. EtOH feeding during post-colitis recovery periods significantly reduced the body weights of mice and the colon lengths, which was effectively blocked by HD5 feeding. Interestingly, HD5 feeding caused a partial prevention of EtOH effect on colitis-induced expression of *Defa4*, *Defa5* and *Defa6* genes in colon. But, in the ileum of EtOH-fed and DSS-treated mice, the expression of *Defa5* and *Defa6* was slightly elevated, whereas the expression of *Defa4* was reduced. This observation suggests that HD5 may have positive influence on the expression of some of the defensin isoforms. EtOH feeding had no effect or minor effect on the levels of mBD-2 and Reg3b mRNA in colon or ileum, but it blocked the colitis-induced expression of these non-Paneth cell defensins, secreted by the surface epithelial cells.

HD5 is a potent antibacterial agent. Therefore, prevention of EtOH and DSS-mediated dysbiosis of intestinal microbiome is likely a mechanism involved in HD5-mediated prevention of recurrent colitis-induced intestinal mucosal damage and its exacerbation by alcohol consumption. To determine whether this potential mechanism is involved in HD5 activity we analyzed dysbiosis by qPCR analysis of major phyla of bacteria that inhabit the colon and bound to mucosal tissue. A significant reduction of overall 16S-rRNA in colonic mucosa may suggest that HD5 feeding reduces the overall binding of microbiota to the colonic mucosa. The combined EtOH feeding and colitis resulted in a dramatic elevation of *Firmicutes* and increase in *Firmicutes*-to-*Bacteriodetes* ratio suggesting that EtOH consumption exacerbates colitis-induced dysbiosis. In addition, our analysis show that EtOH feeding and DSS-induced colitis synergistically elevates *Enterobacteriaceae* bound to colonic mucosa. HD5 treatment very effectively suppresses EtOH and colitis-induced elevation of *Firmicute*s, *Enterobacteriaceae* and *Firmicutes*-to-*Bacteriodetes* ratio in colonic mucosa. These results demonstrate that one potential mechanism involved in HD5-mediated protection of intestinal mucosa from EtOH and colitis is prevention of bacterial dysbiosis in the colonic mucosa.

EtOH feeding and DSS-induced colitis have been associated with the disruption of intestinal epithelial TJ and AJ^21,22^. Immunofluorescence confocal microscopy in the present study indicates that EtOH exacerbates DSS-induced disruption of TJ and AJ. HD5 feeding prevents these effects of EtOH and colitis on TJ and AJ. Prevention of intestinal epithelial junctional disruption was associated with a prevention of EtOH and DSS-induced colitis and mucosal damage. EtOH feeding appears to exacerbate DSS-induced colitis and HD5 effectively blocks these effects of EtOH and colitis. EtOH and colitis together caused loss of goblet cells, architectural distortion, epithelial erosions and crypt drop-out in the colonic mucosa, and HD5 significantly reduced these effects. On the other hand, EtOH or HD5 had no effect on colitis-induced increase in crypt abscess/cryptitis, hyperemia, lamina propria inflammation and mucosal edema. These symptoms are may be unrelated to dysbiosis under the present experimental conditions. Similarly, EtOH feeding synergistically promotes DSS-induced elevation of proinflammatory cytokines such as IL-1β, TNFα and IL-6, and chemokines such as CCL5 and MCP1 in colon. On the other hand, EtOH exacerbates DSS-induced suppression of anti-inflammatory cytokines such as IL-10 and TGFβ. HD5 treatment attenuated these effects of EtOH and DSS-colitis on cytokine and chemokine expression. Similarly, DSS-induced expression of proinflammatory cytokines and chemokines was enhanced by EtOH feeding in the small intestine and HD5 blocked these effects. These results demonstrate the anti-inflammatory effects of orally administered HD5, indicating its potential therapeutic value in the treatment of colitis and EtOH-induced epithelial injury. This observation was confirmed by similar effects of EtOH and colitis on plasma cytokine levels and its prevention by HD5 treatment. Disruption of gut barrier function by EtOH consumption is known to be associated with elevated absorption of bacterial toxins causing endotoxemia^11^. In the present study, we show that HD5 reduced EtOH and colitis-induced elevation of plasma LPS. indicating that HD5 attenuates EtOH and colitis-induced endotoxemia.

In summary, these data demonstrate that alcohol consumption downregulates the expression of antibacterial α-defensins not only in the small intestine, but also in colon induced by colitis. Feeding a human defensin, the HD5, prevents EtOH and colitis-induced disruption of epithelial junctional complexes and inflammation. Attenuation of EtOH and colitis-induced dysbiosis is a potential mechanism involved in the HD5-mediated prevention of epithelial barrier dysfunction, inflammation and mucosal damage. HD5 may have potential therapeutic benefit in the treatment of alcohol and colitis-induced mucosal damage and endotoxin absorption.

### Synopsis

Ethanol down-regulates the expression of α-defensins in the small intestine and colitis-induced expression in the colon in mice. Oral administration of human defensin-5 blocks ethanol and colitis-induced dysbiosis, tight junction disruption and inflammation.

## Electronic supplementary material

Supplementary information
