# Supplementary material for: Human Defensin-5 Blocks Ethanol and Colitis-Induced Dysbiosis, Tight Junction Disruption and Inflammation in Mouse Intestine
Source: Sci Rep. 2018 Nov 2;8:16241. doi: 10.1038/s41598-018-34263-4 (PMC6214960; doi:10.1038/s41598-018-34263-4)
Supplement: Supplementary file 1 — Supplementary information [file 41598_2018_34263_MOESM1_ESM.docx]

Supplemental Information

**Human Defensin-5 Blocks Ethanol and Colitis-Induced Dysbiosis, Tight Junction Disruption and Inflammation in Mouse Intestine**

^1^Pradeep K. Shukla, ^1^Avtar S. Meena, ^1^Vaishnavi Rao, ^1^Roshan G. Rao, ^2^Louisa Balazs, ^1^RadhaKrishna Rao^¶^

Department of ^1^Physiology and Department of ^2^Pathology, University of Tennessee Health Science Center, 3 North Dunlap, Suite S303, Memphis TN 38103

Table S1: Primers used for qPCR

| **Gene** | **5′-3′ Sequence** |
| --- | --- |
| Defcr4 | Forward: CCAGGGGAAGATGACCAGGCTG |
|  | Reverse: TGCAGCGACGATTTCTACAAAGGC |
| Defcr5 | Forward: GCTCCTGCTCAACAATTCTCC |
|  | Reverse: CAGCTGCAGCAGAATACGAA |
| Defcr6 | Forward: GACCAGGCTGTGTCTGTCTC |
|  | Reverse: CCCTTTCTGCAGGTCCCATT |
| IL-1β | Forward: GCAACTGTTCCTGAACTCAACT |
|  | Reverse: ATCTTTTGGGGTCCGTCAACT |
| TNF-α | Forward: CCCTCACACTCAGATCATCTTCT |
|  | Reverse: GCTACGACGTGGGCTACAG |
| IL-6 | Forward: TAGTCCTTCCTACCCCAATTTCC |
|  | Reverse: TTGGTCCTTAGCCACTCCTTC |
| CCL5/RANTES | Forward: GCTGCTTTGCCTACCTCTCC |
|  | Reverse: TCGAGTGACAAACACGACTGC |
| MCP-1/CCL2 | Forward: TTAAAAACCTGGATCGGAACCAA |
|  | Reverse: GCATTAGCTTCAGATTTACGGGT |
| IL-10 | Forward: GCTCTTACTGACTGGCATGAG |
|  | Reverse: CGCAGCTCTAGGAGCATGTG |
| Tgf-β | Forward: CTCCCGTGGCTTCTAGTGC |
|  | Reverse: GCCTTAGTTTGGACAGGATCTG |
| GAPDH | Forward: CTGCACCACCAACTGCTTAG |
|  | Reverse: GGGCCATCCACAGTCTTCT |
| mBD-2 | Forward: CTGCTGCTGATATGCTGCCTC |
|  | Reverse: TAAACTTCCAACAGCTGGAGTGG |
| mBD-3 | Forward: GCTTCAGTCATGAGGATCCATTACCTTC |
|  | Reverse: CGGGATCTTGGTCTTCTCTA |
| Reg3b | Forward: ACTCCCTGAAGAATATACCCTCC |
|  | Reverse: CGCTATTGAGCACAGATACGAG |
| 16S rDNA, (universal) UniF334-F | Forward: ACTCCTACGGGAGGCAGCAGT |
| 16S rDNA, (universal) UniR514 | Reverse: ATTACCGCGGCTGCTGGC |
| 16S rDNA (Firmicutes) 928F-Firm | Forward: TGAAACTYAAAGGAATTGACG |
| 16S rDNA (Firmicutes) Firm1040R | Reverse: ACCATGCACCACCTGTC |
| 16s rDNA (Bacteroidetes) 798cfbF | Forward: CRAACAGGATTAGATACCCT |
| 16s rDNA (Bacteroidetes) cfb967R | Reverse: GGTAAGGTTCCTCGCGTAT |
| 16s rDNA (Actinobacteria) Act920F3 | Forward: TACGGCCGCAAGGCTA |
| 16s rDNA (Actinobacteria) Act1200R | Reverse: TCRTCCCCACCTTCCTCCG |
| 23S rDNA (Enterobacteriaceae) En-lsu-3F 1 | Forward: TGCCGTAACTTCGGGAGAAGGCA |
| 23S rDNA (Enterobacteriaceae) En-lsu-3'R | Reverse: TCAAGGACCAGTGTTCAGTGTC |
| 16S rDNA (E. coli) Ecoli-F | Forward: CATGCCGCGTGTATGAAGAA |
| 16S rDNA (E. coli) Ecoli-R | Reverse: CGGGTAACGTCAATGAGCAAA |


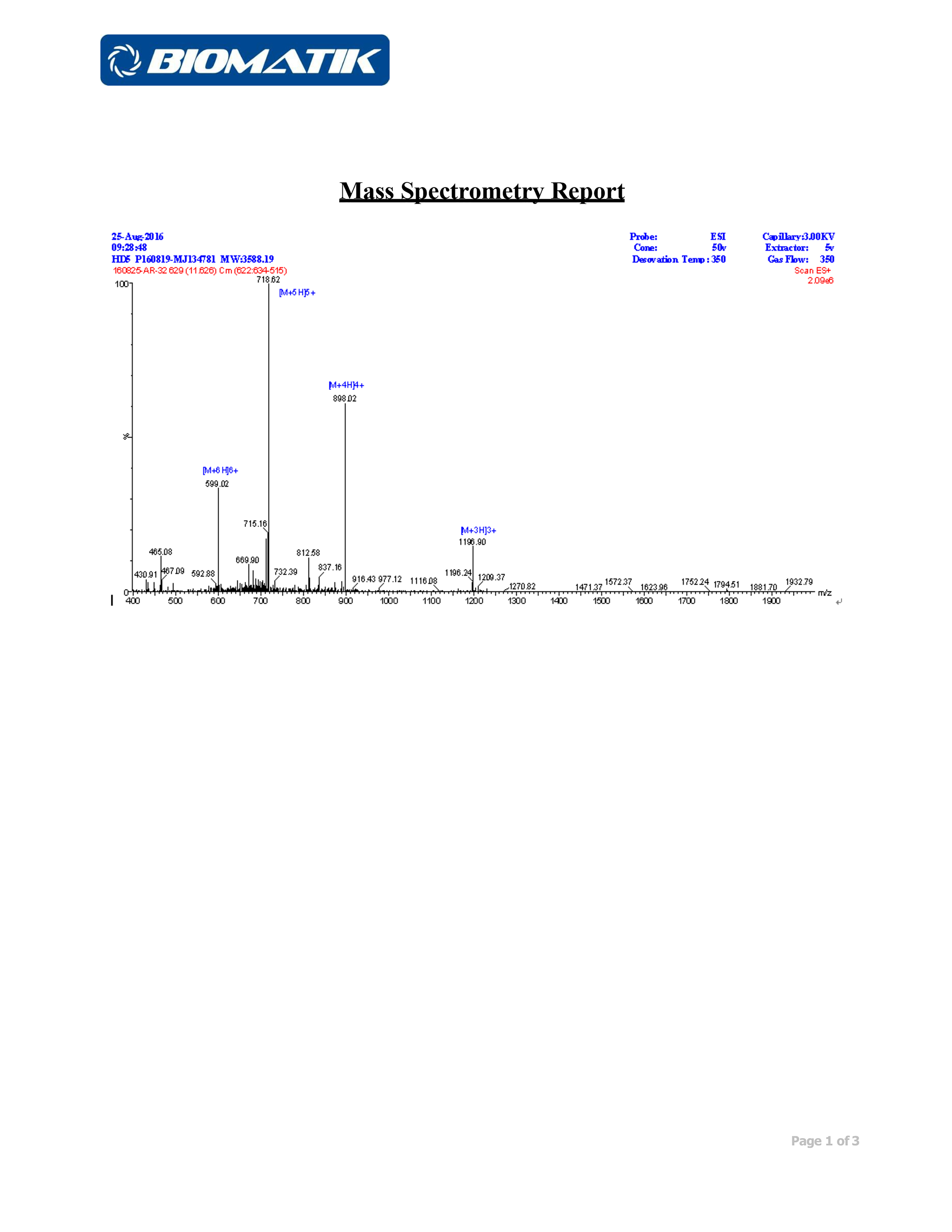


Figure S1: **Mass spectrometry of synthetic HD5: Ion fragmentation data.**

MZ


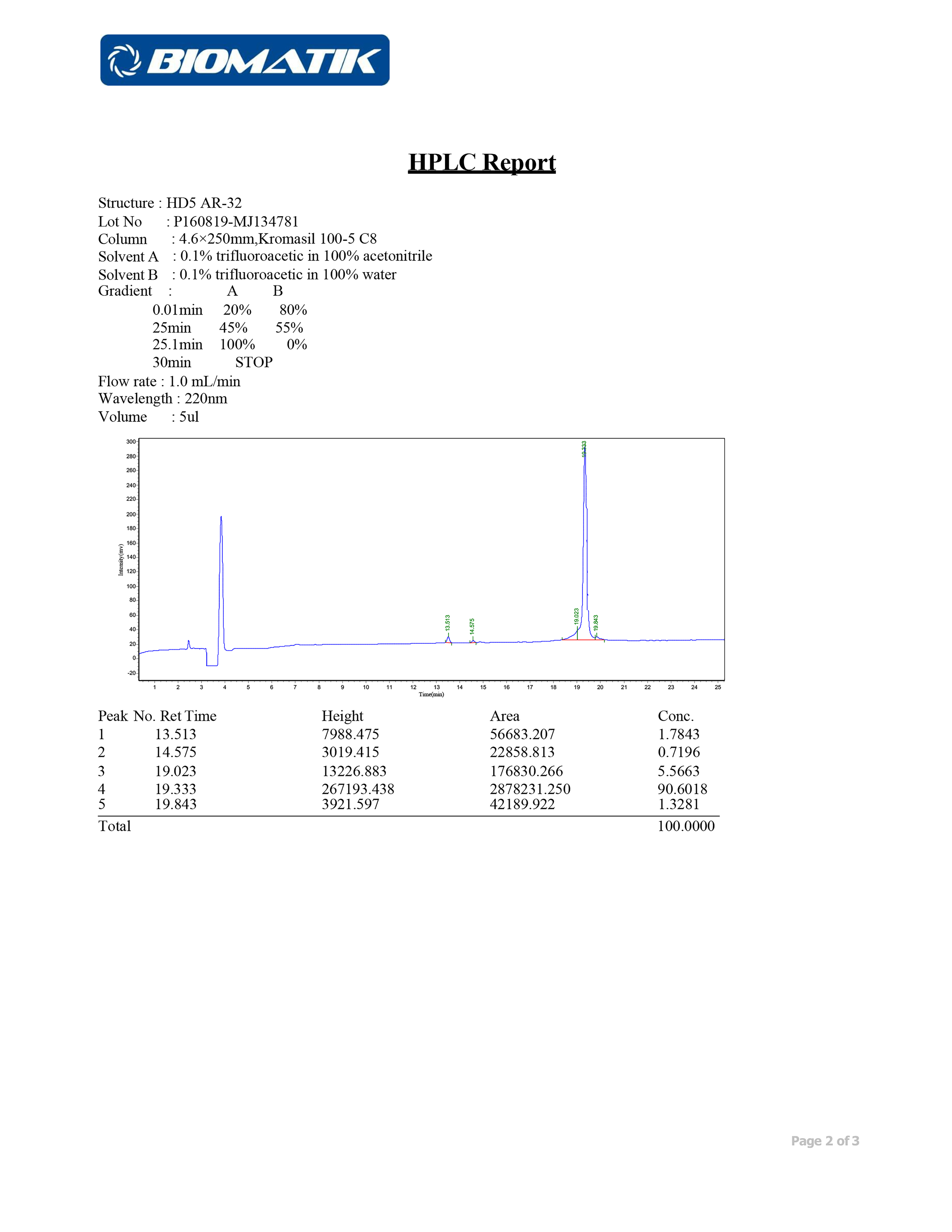


Figure S2: **HPLC purification of synthetic HD5.**
